# Structure of the Vacuolar H^+^-ATPase Rotary Motor Reveals New Mechanistic Insights

**DOI:** 10.1016/j.str.2014.12.016

**Published:** 2015-03-03

**Authors:** Shaun Rawson, Clair Phillips, Markus Huss, Felix Tiburcy, Helmut Wieczorek, John Trinick, Michael A. Harrison, Stephen P. Muench

**Affiliations:** 1 School of Biomedical Sciences, Faculty of Biological Sciences, University of Leeds, Leeds LS2 9JT, UK; 2 Abteilung Tierphysiologie, Fachbereich Biologie/Chemie, Universität Osnabrück, 49069 Osnabrück, Germany; 3 School of Molecular and Cellular Biology, Faculty of Biological Sciences, University of Leeds, Leeds LS2 9JT, UK

## Abstract

Vacuolar H^+^-ATPases are multisubunit complexes that operate with rotary mechanics and are essential for membrane proton transport throughout eukaryotes. Here we report a ∼1 nm resolution reconstruction of a V-ATPase in a different conformational state from that previously reported for a lower-resolution yeast model. The stator network of the V-ATPase (and by implication that of other rotary ATPases) does not change conformation in different catalytic states, and hence must be relatively rigid. We also demonstrate that a conserved bearing in the catalytic domain is electrostatic, contributing to the extraordinarily high efficiency of rotary ATPases. Analysis of the rotor axle/membrane pump interface suggests how rotary ATPases accommodate different *c* ring stoichiometries while maintaining high efficiency. The model provides evidence for a half channel in the proton pump, supporting theoretical models of ion translocation. Our refined model therefore provides new insights into the structure and mechanics of the V-ATPases.

## Highlights

• Subnanometer V-ATPase EM structure gives new insights into mechanism
• Comparison of two distinct catalytic states in a complete rotary ATPase
• Describes a conserved electrostatic bearing that supports high motor efficiency
• Proposes how different *c* ring stoichiometries are accommodated

## Graphical Abstract

**Figure:**
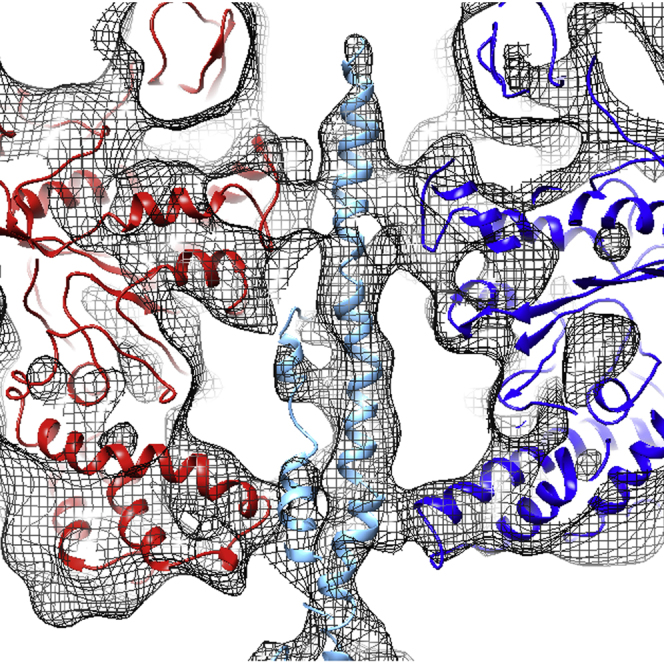

## Teaser

Rawson et al. solve a high-resolution structure of vacuolar ATPase. The complex rests in a catalytic state different from those previously reported. The work gives new insights into the organization, mechanism, and the basis for functional properties such as high thermodynamic efficiency.

## Introduction

The rotary ATPase family includes the F_1_F_o_-ATPase (ATP synthase) of mitochondria, chloroplasts and eubacteria, the vacuolar ATPase (V-ATPase), and the A-ATPases present in archaea and some bacteria (Muench et al., 2011; Marshansky et al., 2014). A common feature of this family is an ATP hydrolyzing/synthesizing motor asymmetrically coupled to a membrane-bound ion pump. In V-ATPases, ATP hydrolysis in the cytoplasmic V_1_ domain drives rotation of a central rotor axle, which transmits torque to the proton pump in the membrane domain (V_o_). Conversely, in F- and A-ATPases operating in synthase mode, torque generated by ion flow through the membrane domain is transmitted to the soluble domain, driving ATP synthesis. Proton translocation is proposed to occur at the interface between rotating and static components in V_o_/F_o_/A_o_, the static part (the stator) forming a continuous structure with the ATP hydrolyzing apparatus. Comparisons of ATP free energy with electrochemical membrane potential or directly measured work output indicate thermodynamic efficiency close to 100% (Junge et al., 2009).

V-ATPases are found in all eukaryotic cells (Forgac, 2007) driving acidification essential to the function of endosomes, lysosomes, and the Golgi apparatus (Marshansky and Futai, 2008). Inhibition blocks endosomal transit and arrests recycling of receptor-ligand complexes. Disease mutations cause dysfunctional glycosylation and aberrant protein sorting by the Golgi (Kornak et al., 2008). The V-ATPase also energizes secondary active transport processes such as neurotransmitter uptake into secretory vesicles (Forgac, 2007). V-ATPases are active at the plasma membrane of some cells, for example, in tumor cells (Sennoune et al., 2004) and in osteoclasts where acid extrusion is essential for resorption of mineralized bone (Blair et al., 1989).

In the V-ATPase V_1_ domain, alternating A and B subunits form a pseudohexameric arrangement, with three catalytic sites located at the B-A interfaces (Muench et al., 2011; Marshansky et al., 2014). ATP hydrolysis moves a helix-loop-helix “lever arm” in subunit A with sequential hydrolysis imposing torque on the central axle comprising the helical coiled-coil D subunit linked at its base to subunit F. The end of the axle is connected to a ring of *c* subunits in the membrane via subunit *d*, allowing concerted *c* ring rotation. The rotor carries ∼35 pN nm of torque (Imamura et al., 2005), which is not rigid and can flex along its length (Song et al., 2013). Proton translocation is thought to occur at the interface between the rotating *c* ring and the static large integral membrane *a* subunit. Models propose that the proton boards the *c* ring via a half channel in *a*, with subsequent stepping of the rotor, allowing proton exit on the opposite side of the membrane via a second channel (Muench et al., 2011). Detailed structural information for the membrane domain of subunit *a* is lacking, although the soluble domain of a bacterial subunit *a* homolog has been solved (Srinivasan et al., 2011). Biochemical studies indicate an N-terminal soluble cytoplasmic domain linked to eight transmembrane helices, with residues in helices 7 and 8 involved in proton movement (Wang et al., 2008). Another small integral membrane subunit, *e*, is poorly characterized, but is reported to be heavily glycosylated and associated with subunit *a* (Merzendorfer et al., 1999).

Futile rotation of *a* with the *c* ring is prevented by the stator attached to the (AB)_3_ motor. In V-ATPase, the stator has three subunit E/G filaments attached to the top of the B subunits and converging with a horizontal collar structure that surrounds most of the midsection of the rotor axle (reviewed in Muench et al., 2011). This collar comprises the *a* subunit soluble domain and subunits C and H. In contrast, the A-ATPase (which lacks C and H) has only two EG filaments (Lau and Rubinstein, 2010), and in F-ATPase the stator is a single filament (Rubinstein et al., 2003). The additional complexity in V-ATPase appears to be an adaptation for more sophisticated control. Low-energy status, such as occurs during larval molt in *Manduca sexta* or as a result of glucose depletion in *Saccharomyces cerevisiae*, causes dissociation of V_1_ from V_o_ in vitro (Sumner et al., 1995; Kane, 1995). Although in vivo experiments suggest a more subtle rearrangement rather than complete separation (Tabke et al., 2014), the functional effects are well defined: catalytic silencing in V_1_ (Parra et al., 2000) and proton impermeability in V_o_ (Beltran and Nelson, 1992). Subunit C may be the receptor for the dissociation signal, and subunit H has been proposed to then prevent ATP cycling by fixing the rotor axle to the stator (Parra et al., 2000; Muench et al., 2013).

Recent technical advances, including more stable microscopes, high-sensitivity direct electron detectors, and new image processing algorithms, have substantially improved the resolution attainable by electron cryomicroscopy (cryo-EM) (Smith and Rubinstein, 2014). Now, cryo-EM of even relatively small and nonsymmetrical membrane protein complexes can resolve structures to below 5 Å (Lu et al., 2014) and conformational changes linked to mechanisms (Cao et al., 2013). In this study, we report the complete structure of a eukaryotic V-ATPase at nanometer resolution, using cryo-EM. Our model provides insights into the organization of its proton-translocating apparatus and the basis for the extraordinarily high efficiency of rotary ATPases. The complex rests in a different catalytic state from previously reported yeast structures, providing new insights into the rotary mechanism.

## Results and Discussion

**Figure 1:**
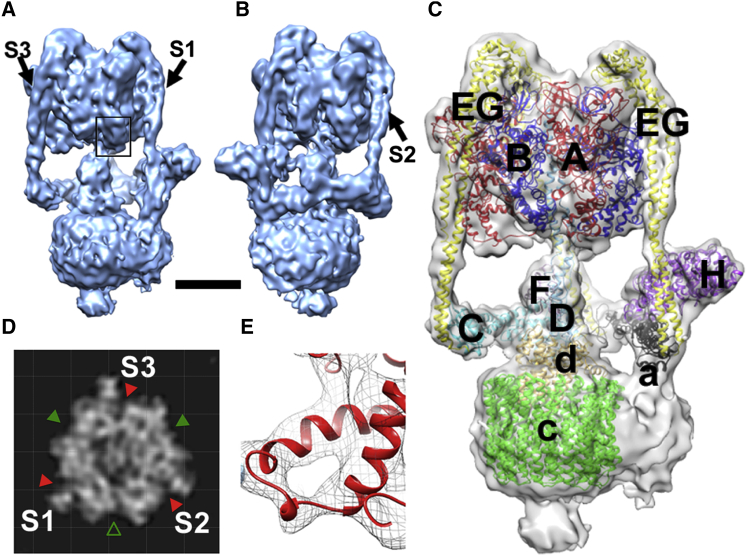
3D Reconstruction of the *Manduca sexta* V-ATPase at ∼1 nm Resolution (A and B) Surface views rotated by 120°, note the open AB site faces the viewer in (B). S1–S3 are stator filaments comprising E and G subunits. Scale bar, 60 Å. (C) Molecular models of *M. sexta* subunits fitted into the reconstruction, based on crystal structures of homologs from *Saccharomyces cerevisiae* V-ATPase or bacterial A-ATPases. See also Movie S1. (D) Section through the electron density map of the V_1_ midsection, with the AB active sites indicated by a green triangle, open site by an open triangle, and the noncatalytic AB interfaces by red triangles. (E) Representative electron density in the V_1_ domain, taken from the square section in (A), around the DELSEED-related region showing the quality of crystal structure fitting.

**Figure 2:**
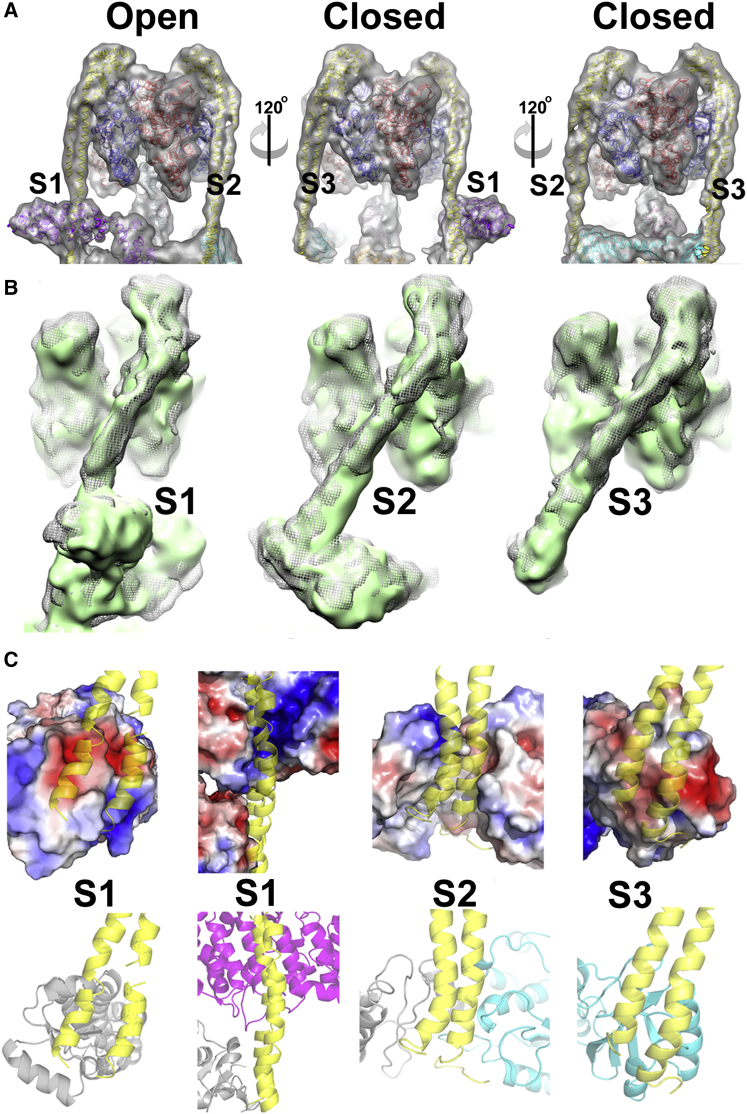
Stator Connections in the V-ATPase (A) Transparent surface view of the V_1_ domain of *M. sexta* V-ATPase with subunits docked, showing the differences between the “open” and “closed” AB domains. Subunits are colored as in Figure 1C. (B) Superposition of stator filaments 1, 2, and 3 of *M. sexta* (mesh) and yeast (green surface) V-ATPase based on a global alignment, showing the very similar EG conformations despite differences in the AB catalytic state. See also Figure S1. (C) Comparisons of the interface for each stator, with electrostatic surface shown above and cartoon representation below. The EG stator, C subunit, H subunit, and *a* subunit are yellow, cyan, magenta, and gray, respectively.

The resolution of the *M*. *sexta* V-ATPase 3D reconstruction, estimated by gold standard 0.143 Fourier shell correlation (Rosenthal et al., 2003), is 9.4 Å (Figure 1; Movie S1). Our previous ∼17 Å model showed overall architecture and subunit organization but did not allow flexible fitting of crystal structures (Muench et al., 2009). Consequently, finer details of subunit organization were not resolved. The nanometer model shows secondary structure, including individual helices, such as those of subunits E and G in the stator filaments (Figures 1A–1E) and the helix-loop-helix torque-generating lever in subunit A (Figure 1E). The active site in the open (unoccupied) conformation is also clearly identifiable (Figures 1B and 1D, and 2). Features of *c* ring and subunit *a* organization in V_o_ are resolved, although individual α helices are not seen, the resolution specifically in the membrane domain being significantly lower than 1 nm (discussed in detail below). For subunit fitting, homology models were generated for all *M. sexta* subunits based on existing crystal structures, with the exception of subunit *a* where sufficient sequence similarity or structural information are not available. Each subunit was flexibly docked using molecular dynamics flexible fitting (MDFF) (Trabuco et al., 2008) giving an optimal fit and, although the resolution does not permit accurate positioning of side chains, the C_α_ positions in the secondary structure elements could be approximated with confidence (Figures 1C and 1E; Movie S1). In the following we focus on aspects of the structure and mechanism not previously discussed because of limited resolution and information about catalytic states.

### Comparing Different Conformational States in V_1_

Examination of V_1_ clearly shows the AB dimer catalytic site in the open state positioned above subunit H between stator filaments 1 (S1) and 2 (S2) (Figures 1 and 2A). That this feature is well resolved and the model is not produced from a ∼1/3 subset of the data strongly suggests that the *M. sexta* V-ATPase predominantly adopts a preferential resting state once separated from the cell. Importantly, in the *Saccharomyces* V-ATPase reconstruction at 11 Å resolution (Benlekbir et al., 2012), the open site is situated above subunit C, between S2 and S3 and 120° rotated from that seen in the *M. sexta* enzyme (equivalent to the site facing the reader in the right-hand image of Figure 2A; see also Figure S1). In the *Thermus thermophilus* A-ATPase, the open site is also situated in a position equivalent to that of the yeast enzyme (Lau and Rubinstein, 2011). Although the reasons for the apparent difference in resting state positions are uncertain, different nucleotide occupancy could be a factor. The purified *M. sexta* enzyme retains ∼0.3 mol of nucleotide phosphate per mol V-ATPase (Huss and Wieczorek, 2007). By inference from crystal structure of bacterial A_1_ (Numoto et al., 2009), any bound nucleotide should be in one or other of the “closed” sites in Figure 2A. However, to our knowledge similar analyses have not been done for other systems, hence meaningful correlations between nucleotide occupancy and the resting state cannot yet be made.

This allows comparison of a whole rotary ATPase motor in two different catalytic states. The most striking feature is that, despite the V-ATPase being arrested at different points in their rotary cycles, the three stator filaments (S1–S3) remain almost completely superimposable when global alignment is done based on subunit *a* (Figure 2B). This shows that catalytic site occupancy and the consequent conformation of the AB domain has no significant effect on the stator filament positions. Instead, stator conformation must be dictated by contacts made with the collar subunits. We previously questioned whether the straighter, apparently more strained conformation of S1 retained even in detached V_1_ is imposed by interaction with subunit H, or by its proximity to the open catalytic site (Muench et al., 2013). Since S1 has essentially the same conformation in both the *M. sexta* and *S. cerevisiae* enzymes, but is only adjacent to the open state in *M. sexta* V-ATPase, the latter possibility can be excluded and interaction with H must impose the straightened conformation on S1. Analysis of the stator interfaces to subunit C (with S2/S3), subunit *a* (S1/S2), and subunit H (S1) shows all three EG subunit pairs make extensive contacts at their N-terminal ends (first 15 residues G, 20 residues E) and that the interface is commonly a charged β-sheet motif (negative for S1 and S3 contacts, mixed for S2; Figure 2C). A significant difference is seen for S1 where an additional interface involving ∼25 residues of subunit E is formed with a highly conserved positively charged patch on subunit H (Figure 2C). Analysis of the residues on S1 forming this interface shows a complementary negative surface highly conserved across 300 sequences examined. The significance of the “deformed” conformation of S1 is unknown.

### An Electrostatic Bearing in the V_1_ Motor

**Figure 3:**
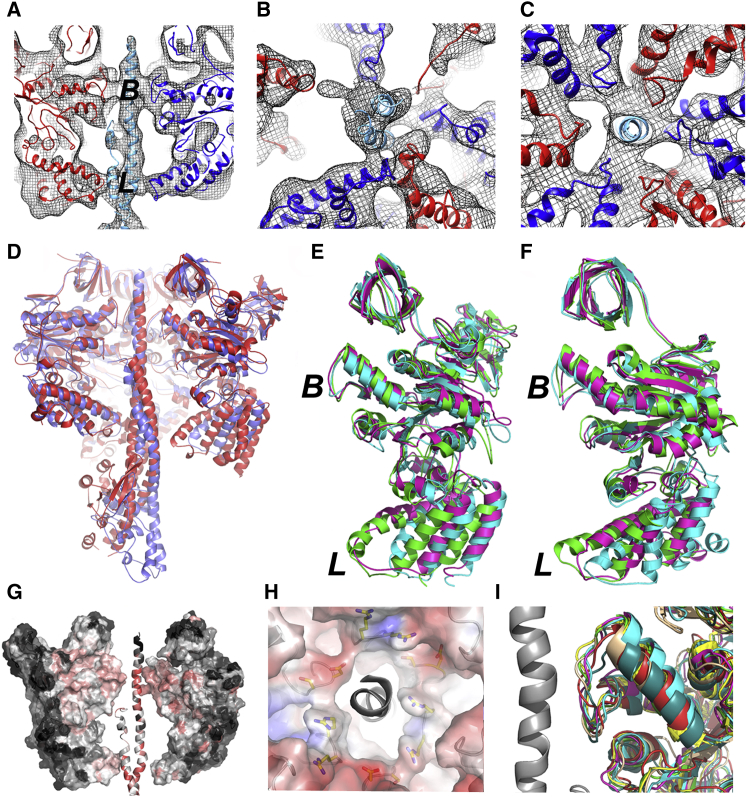
Motor-Axle Interactions in V_1_ (A) Vertical section through V_1_, with the EM map shown in mesh format and models of subunits A (red), B (blue), and D (cyan) fitted. Distinctive contacts can be seen at the lever (*L*) and bearing (*B*) regions. (B) Slice-through of the lever region, and (C) bearing region showing the close packing against the P-^A^/_G_-^D^/_E_-X-G-^Y^/_F_-P (subunit A) and P-^G^/_S_-R-^R^/_K_-G-^Y^/_F_-P (subunit B) loops. (D) Comparison of the *Enterococcus hirae* A_3_B_3_DF crystal structure (red) and *M. sexta* A_3_B_3_DF model (blue). (E and F) Superposition of the open (cyan), loose (green), and tightly bound (magenta) A (E) and B (F) subunits. Greatest differences are at the base region corresponding to the lever arm domain (*L*) involved in torque generation. Little change is observed within the bearing region (*B*). See also Movie S2. (G) Sequence conservation in V_1_, calculated in Consurf (Goldenberg et al., 2009). The continuum ranges from pink (highly conserved) to black (no conservation). Strongest sequence identity is found within the bearing region. (H) Electrostatic surfaces at the bearing region of *M. sexta* V-ATPase shown on a scale of −5.0 (red) to 5.0 (blue). (I) Superposition of the conserved loop region for subunits A (red) and B (blue) from *M. sexta* V-ATPase, A (yellow) and B (green) from *Enterococcus hirae* A-ATPase (Arai et al., 2013), α (cyan) and β (wheat) from bovine mitochondrial F-ATPase (Bowler et al., 2007), and FliI (magenta) from the flagella motor (Imada et al., 2007). The position of the rotor axle subunit D is shown as a gray helix. In (B), (C), and (H) the V-ATPase is viewed from the luminal side and rotation of the axle will be counterclockwise.

F- and A-ATPases share essentially the same rotational mechanism, with their catalytic cycles both operating according to the binding change mechanism (Boyer, 1997). Consistent with this, crystal studies of bacterial A_1_ domain show one empty catalytic site, with two occupied by nucleotide phosphates (Numoto et al., 2009). Structural similarity and directly observed rotational mechanics indicate a similar mechanism for the V-ATPase (Hirata et al., 2003), although crystal structures for eukaryotic V_1_ are not available. Here, the quality of the cryo-EM map allows *M. sexta* homology models to be fitted, identifying points of contact between individual A and B subunits and the rotor axle (Figure 3). Sections through V_1_ parallel to its long axis show the rotor axle passing asymmetrically through the central cavity, making two points of contact to each AB (Figures 3A–3C). The first contact is the loop structures equivalent to the DELSEED “levers” involved in torque generation/transmission in F-ATPase, where clear asymmetry can be seen between the AB domains (Figure 3B). The F subunit orientation with respect to the open catalytic site is the same in both the bacterial A_1_ crystal structure (Arai et al., 2013) and fitted *M. sexta* structure. However, the D subunit fitted to the axle density of the V-ATPase reconstruction has less curvature than the equivalent in A_1_ or DF heterodimer crystal structures, suggesting that it is more constrained when incorporated into the holoenzyme (Figure 3D; Movie S2). Moreover, there are significant differences in AB structures between the A_1_ crystal structure and V_1_ fitted subunits. This may result from differences in the two systems, or from artifacts of crystallizing only partial domains of the complex. Superposition of the fitted AB subunits in the V-ATPase model shows minimal variation between regions nearest the cytoplasmic end of the complex (Figures 3E and 3F), with greatest difference in the lever loops. As in F-ATPase, these loops carry a net negative charge, although a functional role for charge remains uncertain. The lever loop of subunit A contributing to the open catalytic site makes minimal contact with the axle (Figure 3B).

The second region of close contact between (AB)_3_ and the axle occurs ∼40 Å from the cytoplasmic end of V_1_ (Figures 3A and 3C), where A and B subunits both contribute highly conserved loop structures flanked by proline residues: P-^A^/_G_-^D^/_E_-X-G-^Y^/_F_-P in subunit A and P-^G^/_S_-R-^R^/_K_-G-^Y^/_F_-P in subunit B. Analysis of 300 unique A- and V-ATPase sequences shows full conservation of a negatively charged Asp/Glu in subunit A and 100% and 95% conservation, respectively, of the positively charged Arg and Arg/Lys of subunit B (Figures 3G and 3H; Table 1). These loops form a tight hairpin structure, permitted by the fully conserved Pro residues that flank the motifs and the conserved small hydrophobic Ala/Gly and aromatic (Phe/Tyr) residues (Figure 3I). These loop regions show no significant variation in conformation between AB units in open or closed catalytic states (Figures 3C, 3E, and 3F).

Together, they form a hydrophobic collar capped by alternating positive and negative charges (Figure 3H). The presence of a hydrophobic bearing interaction has previously been described for the F-ATPase (Abrahams et al., 1994). Here, we propose an extension to this observation by showing that in the V-ATPase (and A-ATPase relative), this feature has characteristics of an electrostatic bearing. At its point of contact with this feature, the axle D subunit also carries significant charge with a conserved D-E-x-^E^/_D_-R-E-^D^/_E_-F-^F^/_Y_-R-L-K motif (Figure 3G; Table 1). In an environment of opposing charges, the central axle is unable to adopt a fixed stable position, as there are no local extremes of position. This bearing would therefore constrain the axle but could also provide an essentially frictionless interaction, consistent with the extraordinarily high efficiency of the motor (Junge et al., 2009). Constraint by the bearing could also contribute to keeping the foot of the D subunit engaged with subunit *d*, which couples the axle to the *c* ring. D-*d* coupling is predicted by fluctuating finite element analysis modeling to be relatively loose (Richardson et al., 2014) and may play a role in the structural rearrangements associated with ATP silencing in the absence of glucose (Sumner et al., 1995; Kane, 1995; Tabke et al., 2014). F-ATPase shows a similar charged feature in the motor domain with highly conserved PGREAYP (α subunit) and PSAVGYQP (β subunit) motifs (Table 2). This allows for one hydrophobic interface and one interface with alternative positive and negative charge. The γ subunit axle in F-ATPase shows a more uniformly hydrophobic surface than the equivalent feature in the V/A-ATPases. FliI, an ATPase involved in protein export during assembly of the flagellar motor, also contains a similar motif (Table 2), suggesting a common function. Moreover, superposition of the loop structures from A/F/V-ATPase and FliI show a highly conserved architecture (Figure 3I). It is important to note that by placing opposing charges adjacent to each other, their net charge may be neutral. However, V-ATPase has alternating charges, with ∼95% sequence identity for the positively charged residues in subunit B perhaps not counterbalanced in subunit A, with only 44% sequence identity of two negatively charged residues.

### Mechanical Coupling in the Rotor Axle

**Figure 4:**
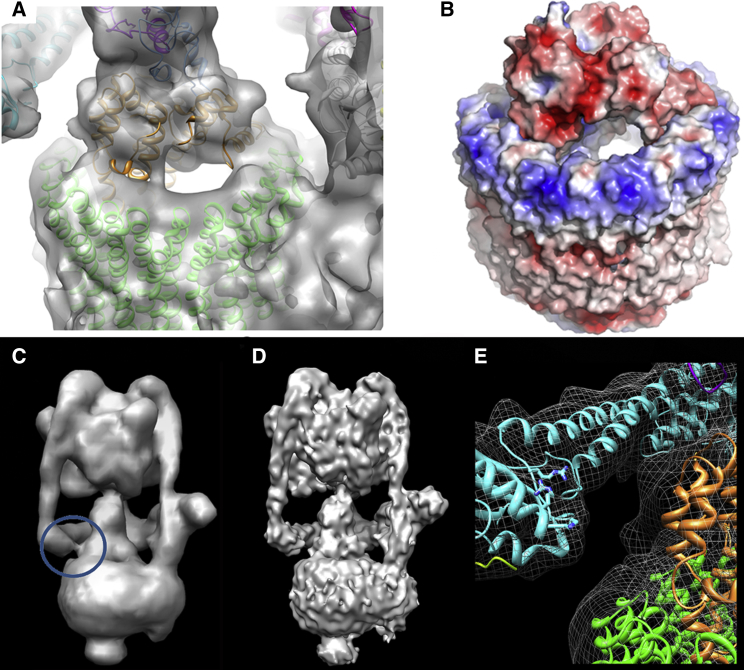
Rotor Coupling in the V-ATPase (A) *M. sexta* map around the *c* ring/subunit *d* connection. Significant cavities between the subunits are evident. (B) Electrostatics of the *c* ring/*d* subunit interface shows the strong net negative charge of *d* complementing the positive charge of the *c* ring. Electrostatics were calculated and scaled as detailed in Figure 3. Note that both (A) and (B) are in approximately the same orientation. (C and D) Apparent contact between subunits C and *d* in the *M. sexta* reconstructions at 11 Å (C) and 9.4 Å (D). The “linker” region is circled. (E) Cartoon of subunits C (cyan), *d* (orange), and the *c* ring (green). Residues making a positive surface are in stick format and are 99% (Arg248), 87% (Lys244), and 95% (Arg241) conserved at that position, based on a 200-sequence comparison using Consurf (Goldenberg et al., 2009).

An unexplained feature of rotary ATPases is their variable *c* ring stoichiometry while maintaining a similar overall axle architecture and, presumably, mechanism. Our V-ATPase reconstruction shows that the *d* subunit makes surprisingly little contact with the *c* ring “socket” (Figure 4A). Large cavities restrict contact to three distinct points at the rim of the *c* ring, limiting the *d* subunit/*c* ring interface. Despite the apparently small contact, torque transmission between axle and *c* ring is highly efficient. Instead of tightly plugging the *c* ring and acting as a direct-drive coupling, we suggest that *d* functions as a rotating lever arm acting on the rim of the *c* ring, a system better adapted to different *c* ring stoichiometries and hence different diameters (Vollmar et al., 2009; Meier et al., 2005; Pogoryelov et al., 2005). It is interesting to note that a similar level of interaction has been seen in the comparable interface in the *T. thermophilus* A-ATPase (Lau and Rubinstein, 2011). This would suggest a conserved mechanism of torque delivery across the A/V-ATPase family, which have both been shown have varying *c* ring stoichiometries.

Electrostatics may also contribute to *d*/*c* ring coupling: The *c* ring interface has a band of highly conserved positive charge complemented by the overall negative charge of subunit *d* (Figure 4B). A conserved feature in all published A/V-ATPase reconstructions is a density that apparently connects the final stator (S3 in V-ATPase and S2 in A-ATPase) to subunit *d* (Figure 4C). Anomalously, this would hinder the free rotation of the axle relative to the stator network, which in molecular dynamics (MD) simulations increases complex stability (Richardson et al., 2014). At ∼1 nm resolution this feature is weaker, indicative of a close but crucially noncontacting surface (Figure 4D). More accurate subunit C fitting permitted by improved resolution shows that this linker is formed by conserved positively charged residues close to the predominantly negatively charged *d* subunit (Figure 4E). This may play a role forming the uniform and stable ground state observed in the V-ATPase.

### Proton Pump Organization in V_o_

**Figure 5:**
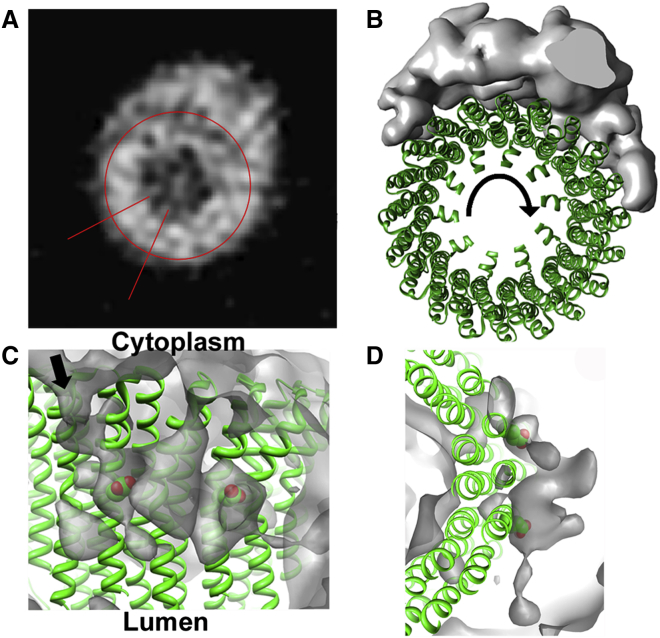
Organization of V_o_ (A) Section through the V_o_ region of the map. The red circle shows the approximate boundary of the *c* ring and lines delineate a four-helix bundle of one *c* subunit. (B) Segmentation of the map showing the extent of subunit *a* around the *c* ring (represented by the NtpK 10-mer structure (PDB ID 2BL2; green). Arrow donates direction of rotation. (C and D) Region of low-density “cavities,” enclosed within the gray surface, at the *c* ring (green)/subunit *a* interface consistent with a proton-accessible “half channel” (arrow). The conserved glutamate in helix 4 of subunit *c* playing a key role in proton transfer is shown in stick format. This occurs at the same depth in the membrane as the low-density feature, which is at a position appropriate for the previously hypothetical “proton half channel,” discussed in the text. Views are parallel to the plane of the membrane (C) and from the luminal side (D).

The connection between subunit *a* and the *c* ring is a crucial part of rotary ATPases but is the poorest resolved of all the subunit interfaces. Although global resolution in our model is ∼1 nm, V_1_ is significantly more detailed than V_o_. Helical segments in V_o_ could not be assigned, since resolution that was significantly worse than 1 nm left them completely unresolved. We speculate that this may be due to conformational heterogeneity in the proton pump region of the complex. However, a number of important V_o_ features are visible. The region corresponding to the *c* ring surrounds an area of relatively low density that contains electron-dense regions (Figure 5A). These could represent bound lipids (Zhou et al., 2011) that may function to maintain membrane coupling, but could also include some protein, discussed below. In the segment of the *c* ring furthest from the asymmetric mass that is subunit *a*, several adjacent ∼10 Å diameter objects are visible, which most likely are the four transmembrane helices of each subunit *c*. These are not resolved near subunit *a*, indicating variability in the local environment. Each putative helix in the *c* ring forms ∼22° of the circle, suggesting a complete ring contains 16 helices. Assuming the same organization as in the *Enterococcus hirae* NtpK ring (Murata et al., 2005), this suggests an 8-mer as the most likely ring stoichiometry in *Manduca*.

A second striking V_o_ feature is the extent to which the *c* ring is covered by subunit *a*. In A-ATPase, subunit *a* extends around ∼50° of the *c* ring (Lau and Rubinstein, 2010), but in the *M. sexta* V-ATPase this extends to as much as 160° (Figures 5A and 5B). This feature is compatible with studies on the V-ATPase inhibitor PA1b, which only bound to a limited number of *c* subunits across an arc of ∼120° (Muench et al., 2014). Subunit *a* hence acts as an incomplete sleeve that partially envelopes the *c* ring. It is noted that the A-ATPase (Lau and Rubinstein, 2011) and V-ATPase (Benlekbir et al., 2012) structures, which have been stabilized by dodecyl maltoside, contain a strong band of density around the *c* ring which is reported to be detergent. This feature is not present in our structure where C_12_E_10_ was used, and likely reflects differences in detergent properties. Most importantly, the *M. sexta* enzyme is still highly active in this detergent.

Current models of proton translocation envisage discontinuous “half channels” that allow ions to board and disembark the *c* ring rotor as it rotates through the interface with subunit *a* (Gräbe et al., 2000; Junge et al., 1997). A universally conserved glutamate residue in helix 4 of subunit *c* is required for ion translocation, and its carboxyl group carries the proton (Noumi et al., 1991). Similarly, highly conserved arginine residues on putative transmembrane helices 7 and 8 of subunit *a* are required for function (Forgac 2007; Toei et al., 2011). In F-ATPase, site-directed labeling data point to an aqueous pore in subunit *a* (Angevine et al., 2007), but direct structural evidence consistent with a pore or channel in V-ATPase has not been presented. Our reconstruction and sections through the model show a region of low density against the *c* ring formed by subunit *a* and accessible from the cytoplasmic side (Figures 5C and 5D). The depth of this feature and its volume make it capable of interacting with two of the key subunit *c* Glu residues involved in proton translocation, a requirement for proton loading onto the *c* ring rotor. The region of subunit *a* likely to be adjacent to this pore is the putative helix 7 (Kawasaki-Nishi et al., 2003). The position and size of the low-density feature are consistent with the entry “half channel” proposed in models of ion translocation (Junge et al., 1997), but higher resolution will be required to validate this. In the *T. thermophilus* A-ATPase (Lau and Rubinstein, 2011), the proton entry/exit “half channels” are assigned to two widely spaced interhelical cavities within the *a* subunit. In our model, the cytoplasm-accessible “half channel” is much closer to the edge of the *a*-*c* ring interface at which the *c* ring rotor enters. To provide the anionic “acceptor” for a cytoplasmic proton, the proton being pumped to the luminal side would need to disembark the *c* ring as it enters the subunit *a*-*c* ring interface. Consequently, the hypothetical exit and entry “half channels” would need to be relatively close together, consistent with the model proposed by Michael Forgac and coworkers (Toei et al., 2011). In this model (based on chemical probe accessibility, crosslinking, and mutagenesis data), the conceptual “half channels” involve essential Arg residues contributed by adjacent subunit *a* helices 7 and 8, and hence are physically very close.

**Figure 6:**
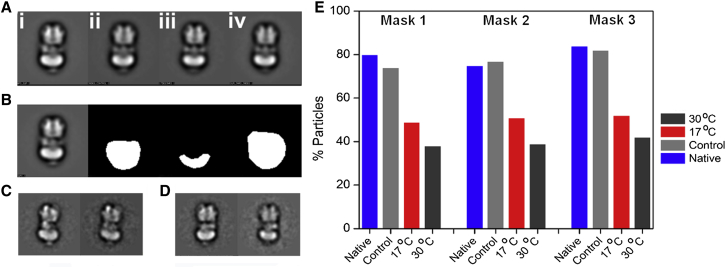
Deglycosylation Analysis of *M. sexta* V-ATPase (A) Image sums of the full data sets from V-ATPase (i) in the native untreated state (V-ATPase^n^), (ii) treated as for deglycosylated enzyme but lacking PNGase F (V-ATPase^c^), (iii) and (iv) treated with PNGase F at 17°C (V-ATPase^d17^) or 30°C (V-ATPase^d30^), respectively. (B) Image sum and masks used. (C and D) Comparable classes from the control (left) and deglycosylated sample (right) of two different side views. (E) Percentage of particles displaying density at the base of V_o_ in the native, control, and PNGase-incubated V-ATPase (with treatments at 17°C and 30°C). Each data set was processed with the three different masks shown in (B) to check for processing artifacts.

A previously unassigned feature of V-ATPases is the ∼40 Å diameter density protruding from the luminal surface of V_o_, giving it a bowl-like appearance (Figures 1A and 1B). This density has been proposed to be accessory subunit Ac45, based on its presence in EM images of mammalian V-ATPases and absence in the *Saccharomyces* complex, which lacks an Ac45 homolog (Wilkens et al., 1999). Deglycosylation of the *M. sexta* enzyme with PNGase F under conditions that maintain the enzyme in the native state removed a significant proportion of this density (Figure 6), indicating that at least a major proportion of it is polysaccharide. Candidates for the V_o_ glycoprotein are Ac45 and subunit *e*, and the position of the density directly below the *c* ring making it unlikely to be subunit *a*. *M. sexta* has a gene for an Ac45 homolog, but extensive mass spectrometry analysis of V-ATPase purified from the midgut of *M. sexta* points to it being absent from the purified enzyme, with all other subunits being identified. The density therefore most likely indicates the location of the heavily glycosylated subunit *e* at the base of the *c* ring, a position consistent with its binding to extracellular PA1b toxin (Muench et al., 2014). It follows from this that although some of the density observed within the interior of the *c* ring (Figure 5A) may be bound lipid (Zhou et al., 2011), the two predicted helices of subunit *e* may also contribute.

### Conclusions

This is the highest-resolution model of a complete V-ATPase complex, revealing insights into its structure and mechanism. A significant obstacle to understanding the changes that accompany catalytic cycling in rotary ATPases has been the inability to trap different states in the full complex. Due to the speed of the V-ATPase catalytic cycle and the currently limiting freezing speeds of vitrification apparatus, these states cannot be simply trapped. There is a clear difference in the position of the open site within the current *M. sexta* and yeast V-ATPase (Benlekbir et al., 2012). Importantly, the two reconstructions show remarkably similar global architectures. In particular, the stator filament shapes do not adapt to reflect the different nucleotide occupancy states of the AB domains. Rather, interactions made to the collar *a*, C, and H subunits alone must dictate different stator conformations. The extensive contact made between subunit H and S1 likely causes the much straighter conformation of this EG dimer, the significance of which is yet to be determined. Previous work has suggested that the V-ATPase undergoes significant flexing during the ATPase cycle (Song et al., 2013; Stewart et al., 2012). The inherent plasticity shown by the EG stators, with their ability to adopt three distinct conformations, may still permit the apparent ∼7° flexing seen in EM, MD simulations, and crystal structures (Song et al., 2013; Stewart et al., 2012; Giraud et al., 2012). Importantly, they must relax back to the same state after ATP cycling rather than retaining new conformations.

Our model shows contacts between the central rotor axle and AB domains. In addition to the well-characterized lever arm region, the rotor axle contacts a density that, although hydrophobic along its equatorial region, shows a clear arrangement of alternating charges. These charged residues are highly conserved, as are the complementary charged residues adjacent to them on the rotor axle. We suggest that this results in a frictionless electrostatic bearing whereby the central rotor axle is stabilized by charge interaction/repulsion rather than by van der Waal interactions, which could be a key factor in the remarkably high efficiency of the rotary ATPases.

A major question about rotary ATPases has been how the same basic rotor architecture can accommodate widely varying *c* ring stoichiometries. Here we show that the *d* subunit/*c* ring interface is considerably smaller than previously described in the V-ATPase, relying on electrostatic interactions, contacting only the rim of the *c* ring rather than comprehensive shape complementarity. This allows subunit *d* to act more like a guide wheel, which would tolerate differing *c* ring sizes while still delivering torque.

Within subunit *a* is a region of low density whose size and position is consistent with the presence of a proton half channel, providing structural evidence in support of theoretical models of ion translocation. The position of subunit *e* has been uncertain, but it is likely that it is contained within the *c* ring, perhaps playing a role in preventing proton leakage.

Our model reveals key insights into the mechanics and structure of the *M. sexta* V-ATPase and the rotary ATPase family in general. Significant questions remain, but the model is an important step toward understanding this ubiquitous and biologically important family of rotary motors. Continued improvements in direct detectors and processing algorithms for cryo-EM are likely and will further improve resolution, helping to provide answers to these questions.

## Experimental Procedures

**Figure 7:**
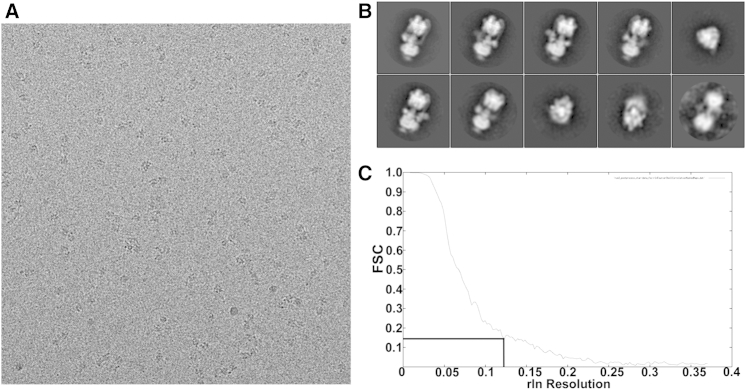
Data Processing of *M. sexta* V-ATPase (A) Representative cryo-EM micrograph of *M. sexta* V-ATPase on a 10 nm thick carbon support film. (B) Reference-free classes generated in RELION; note the clarity of the stator connections. (C) FSC plot for the resulting RELION reconstruction.

*M. sexta* V-ATPase was extracted and purified and its activity tested as previously described (Huss et al., 2002). Sample quality was checked by negative stain microscopy, revealing a monodisperse sample. Cryo-EM grids were prepared using a Vitrobot mark IV (7.5 s blot time) and Quantifoil 400 mesh grids with a 10 nm carbon support layer to improve sample distribution. Data were collected on a Titan-Krios microscope, operating at 300 kV and fitted with a back-thinned FEI Falcon II direct detector. A total of 1366 micrographs were collected using a calibrated magnification of 103,704 (Å/pixel value of 1.35). Data were collected automatically using EPU software and a defocus range from 1.7 to 5.5 μm, determined by CTFFIND3 (Mindell and Grigorieff, 2003). Particles were handpicked using EMAN2 (Tang et al., 2007) resulting in 30,730 particles, which were subject to reference-free classification in RELION 1.3 (Scheres, 2012) and IMAGIC-5 (van Heel et al., 1996) (Figure 7). Particles which populated poorly defined classes or were representative of dissociated V_1_ or V_o_ were removed in iterative rounds of 2D classification in RELION, resulting in 7,160 particles that produced a ∼12 Å reconstruction in autorefinement. Particles with the lowest log-likelihood score were removed, resulting in 6,714 “good” particles and improved resolution to 11 Å. Further improvements in the model were obtained by movie correction (5-frame running average) resulting in a final 9.4 Å resolution model, calculated by the gold standard Fourier shell correlation (Rosenthal et al., 2003) (Figure 7C). 3D classification of the data failed to produce any reconstructions in alternative catalytic states, regardless of the particle stack used.

Subunit fitting was done in Chimera to obtain crude orientation, followed by MDFF (Trabuco et al., 2008). Significant deviations from their original crystal structures were required to fit A, B, H, E, G, and D into the EM map, with only minor deviations required for subunits C, D, F, *c*, and the soluble region of *a*. This is often indicative of crystal structures of subunits of large complexes where these have been solved without the natural constraints normally provided by neighboring subunits. However, this does not diminish the information these crystal structures provide, but rather adds to information about their mechanical properties. Homology models were generated using the PHYRE2 webserver (Kelley and Sternberg, 2009), giving high-confidence models based on the close sequence similarities between the *M. sexta* and template structures. *M. sexta* homolog subunits are based on the crystal structures from A, B, D (Numoto et al., 2009), F (Makiyio et al., 2005), C (Drory et al., 2004), EG (Oot et al., 2012), *d* (Iwata et al., 2004), H (Sagermann et al., 2001), *c* (Murata et al., 2005), *a* (Srinivasan et al., 2011). Sequence homology matching used Consurf (Goldenberg et al., 2009) searching for 300 sequences with between 20% and 90% sequence identity.

### Deglycosylation of the *M. sexta* V-ATPase

Purified *M. sexta* V-ATPase (10 μg; V-ATPase^n^) was incubated with 500 units of peptide *N*-glycosidase F (PNGase F, New England Biolabs) in 50 mM sodium phosphate buffer (pH 7.5) with 100 μM AEBSF, 30 nM aprotinin, 200 nM E-64, and 200 nM leupeptin (Calbiochem) for 3 h at 17°C (V-ATPase^d17^) and 30°C (V-ATPase^d30^). Control samples (V-ATPase^c^) were incubated in the same buffer with protease inhibitors but without PNGase F.

### Mass Spectroscopy

Control and deglycosylated V-ATPase were overloaded onto an SDS-PAGE gel and bands covering 30–50 kDa were removed and digested using trypsin solution. Liquid chromatography-tandem mass spectrometry (LS-MS/MS) analysis was performed on an Ultimate 3000 nano LC system (Dionex, Amsterdam, the Netherlands). The column eluant was directly interfaced to a quadrupole-ion mobility orthogonal time-of-flight (TOF) mass spectrometer (Synapt HDMS; Waters UK, Manchester) via a Z-spray nanoflow electrospray source. The MS was operated in positive TOF mode using a capillary voltage of 3.2 kV, cone voltage of 25 V, backing pressure of 2.47 mbar, and a trap bias of 4 V, with source temperature of 80°C and argon used as the buffer gas at 5.0 × 10^−4^ mbar. Data acquisition used data-dependent analysis with a 1-s MS over *m/z* 350–3000 followed by three 1 s MS/MS taken of the three most intense MS-spectrum ions. Data processing was performed using the MassLynx v4.1 suite of software. Peptide MS/MS data were processed with ProteinLynx Global Server (Waters) and searched against UniProtKB/SwissProt database (release 2011_12) and the *M. sexta* Ac45 sequence.

### Negative Stain Electron Microscopy

Stained grids were produced by placing ∼3 μl of protein (∼50 μg/ml) onto glow-discharged carbon-coated grids followed by three droplets of 1% uranyl acetate. Four sets of grids were made: V-ATPase^n^ (native), V-ATPase^c^ (control with PNGase buffer but no enzyme), V-ATPase^d17^ (deglycosylated at 17°C), and V-ATPase^d30^ (deglycosylated at 30°C). Grids were examined on a FEI Tecnai T12 microscope fitted with a LaB_6_ filament, operating at 120 kV. Micrographs were recorded with a defocus between −0.8 and 1.5 μm on a Gatan Ultrascan 2Kx2K CCD camera at 23,000× magnification resulting in 4.4 Å/pixel. Micrographs were picked using PARTICLE (www.image-analysis.net/EM) generating image stacks for each experimental condition. IMAGIC-5 (van Heel et al., 1996) was used to normalize and band-pass filter the data to remove low (<0.075 Å^-1^) and high (>0.45 Å^-1^) spatial frequencies. A circular mask was applied to remove excess background noise. All data were aligned in IMAGIC-5 (van Heel et al., 1996) against the same set of reprojections from the cryo-EM *M. sexta* V-ATPase (Muench et al., 2009) filtered to 30 Å resolution. The data were classified using multivariate statistical analysis (MSA) and hierarchical ascendant classification. This used a mask that covered the full molecule, with classes that aligned poorly, showed dissociated particles, or were unstable during processing being removed. Particle numbers in the V-ATPase^n^, V-ATPase^c^, V-ATPase^d17^, and V-ATPase^d30^ samples were 4,909, 3,105, 3,578, and 3,592, respectively. Each aligned image stack was then classified three times using masks 1–3 (Figure 6) into 30 and 50 classes which would contain 50 particles if the data classified evenly (100, 62, 73, and 72 classes for the V-ATPase^n^, V-ATPase^c^, V-ATPase^d17^, and V-ATPase^d30^ data, respectively). For each data set particles in classes containing clear density, no density, or ambiguous density were separated and counted.

## Author Contributions

S.R. and S.P.M. conducted EM data processing and analysis. C.P., M.H., F.T., and M.A.H. carried out biochemical analysis and sample preparation. S.R., M.H., H.W., J.T., M.A.H., and S.P.M. designed experimental procedures. S.R., H.W., J.T., M.A.H., and S.P.M. analyzed results and wrote the manuscript.

## Figures and Tables

**Table 1:** Sequence Conservation of the PADEGYP and PGRRGYP Motifs in the A/V-ATPase A and B Subunits, Respectively, and DEX^E^/_D_RE^D^/_E_F^F^/_Y_RLK Motif in Subunit D

A/V-ATPase Subunit A PADEGYP Motif	A/V-ATPase Subunit B PGRRGYP Motif	A/V-ATPase Subunit D DEX^E^/_D_RE^D^/_E_F^F^/_Y_RXK
P(100)	P(99.7) A(0.3)	D(96.5) E(3) G(0.5)
A(59.7) G(40.3)	G(94.6) S(5.4)	E(100)
D(65) E(35)	R(100)	X
E(44) S(36.7) Q(14.3) A(4) G(1) N(0.3) Y(0.3)	R(84.3) K(12.4) Q(2.3) G(1)	E(61) D(38) S(0.5) A(0.5)
G(100)	G(99) S(1)	R(100)
Y(84) F(16)	Y(85.6) F(14.4)	E(99.5) Q(0.5)
P(100)	P(100)	E(81) D(18) N(0.5) R(0.5)
		F(99) I(0.5) C(0.5)
		F(48.8) Y(42) T(4.2) V(4)
		R(100)
		L(95) M(2.5) I(2.5)
		K(99.5) M(0.5)

The conservation at each position is based on alignment of 300 sequences and is shown in parentheses as a percentage.

**Table 2:** Sequence Analysis of V-ATPase A/B Subunit Homologs in the F-ATPase and Flagellar FliI Motor Proteins

Flagellar FliI Subunit PATKGYTP Motif	F-ATPase α-Subunit PGREAYP Motif	F-ATPase β-Subunit PSAVGYQP Motif
P(100)	P(100)	P(99.7) T(0.3)
A(63) T(24.3) V(12.3) I(0.3)	G(100)	S(100)
T(88.7) S(7.6) Q(2.7) A(0.7) M(0.3)	R(100)	A(99.7) S(0.3)
K(73.3) R(26.7)	E(100)	V(100)
G(100)	A(100)	G(100)
Y(99) F(1)	Y(92.4)F(7.6)	Y(100)
T(50.3) P(49.7)	P(100)	Q(100)
P(100)		P(100)

Conservation at each position is shown in parentheses as a percentage.

## Supplemental Information

Document S1. Figure S1

Movie S1 (related to Figure 1). Cryo-EM Analysis of the V-ATPaseThe M. sexta V-ATPase reconstruction with subunits fitted and labeled

Movie S2 (related to Figure 2). Structural Comparison between the A. hirae A1 and M. sexta V1 DomainThe two structures are morphed starting from the A. hirae structure and finishing with the M. sexta structure. Note the straightening of the central axle in the later structure

Document S2. Article plus Supplemental Information
