# Supplementary material for: Structure of the Vacuolar H+-ATPase Rotary Motor Reveals New Mechanistic Insights
Source: Structure. 2015 Mar 3;23(3):461–71. doi: 10.1016/j.str.2014.12.016 (PMC4353692; doi:10.1016/j.str.2014.12.016)

Structure, Volume *23*

Supplemental Information

Structure of the Vacuolar H^+-ATPase Rotary Motor Reveals New Mechanistic Insights

Shaun Rawson, Clair Phillips, Markus Huss, Felix Tiburcy, Helmut Wieczorek, John Trinick, Michael A. Harrison, and Stephen P. Muench

Supplementary Movie 1 (related to Figure 1): cryo-EM analysis of the V-ATPase. The *M. Sexta* V-ATPase reconstruction with subunits fitted and labelled.

Supplementary Movie 2 (related to Figure 2): structural comparison between the *A. hirae* A_1 and *M. Sexta* V_1 domain. The two structures are morphed starting from the *A. hirae* structure and finishes with the *M. sexta* structure. Note the straightening of the central axle in the later structure.

Supplementary Figure 1 (related to Figure 2): analysis of the V-ATPase cryo-EM reconstructions from *M. sexta* and Yeast. Comparison between the *M. sexta* (grey surface, top row) and yeast (green surface, bottom row) V-ATPase reconstructions. Each catalytic AB domain is shown with the open site clearly seen between stators S1 and S2 in *M. sexta* and S2 and S3 in yeast. Figure S1 is related to Figure 2.

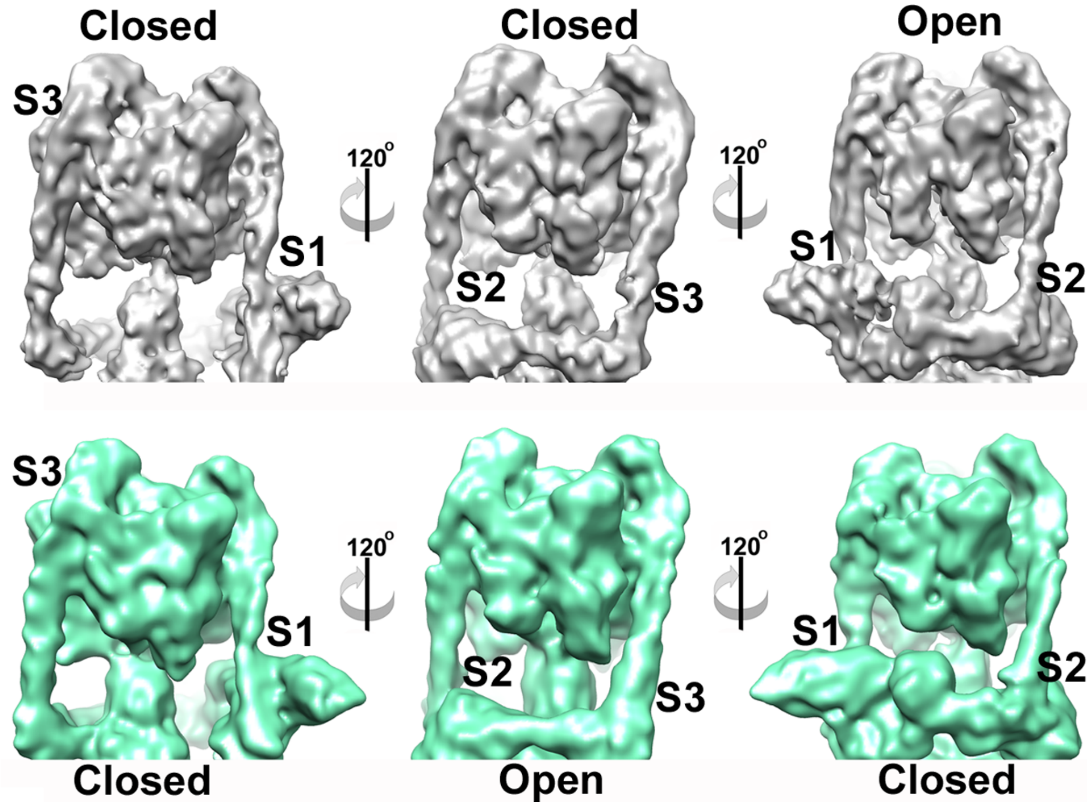

Supplement: Document S1. Figure S1 [file mmc1.pdf]
