# Supplementary figures and images for: Structure of the Vacuolar H+-ATPase Rotary Motor Reveals New Mechanistic Insights
Source: Structure. 2015 Mar 3;23(3):461–71. doi: 10.1016/j.str.2014.12.016 (PMC4353692; doi:10.1016/j.str.2014.12.016)

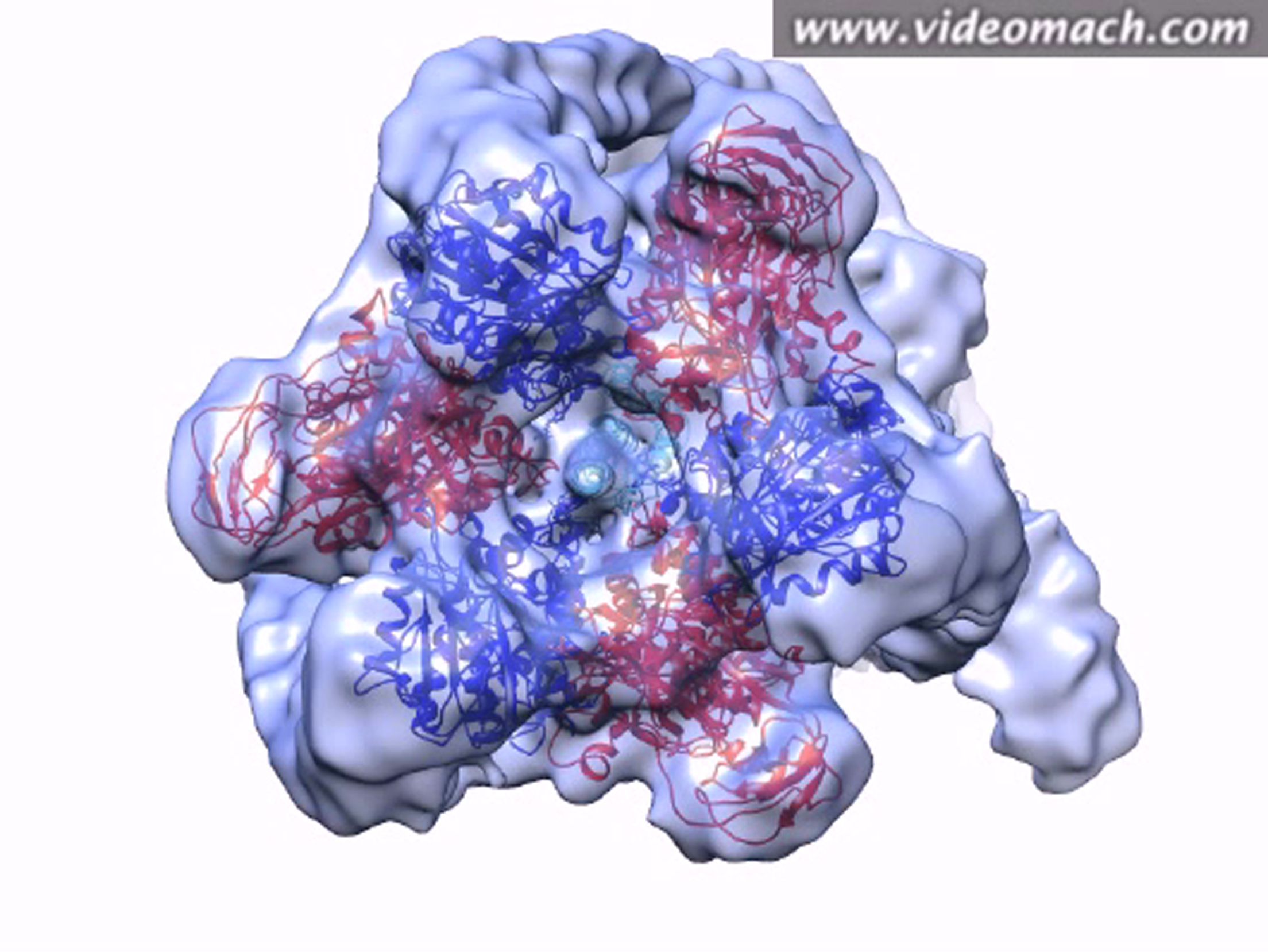

Supplement: Movie S1 (related to Figure 1). Cryo-EM Analysis of the V-ATPaseThe M. sexta V-ATPase reconstruction with subunits fitted and labeled [file mmc2.jpg]

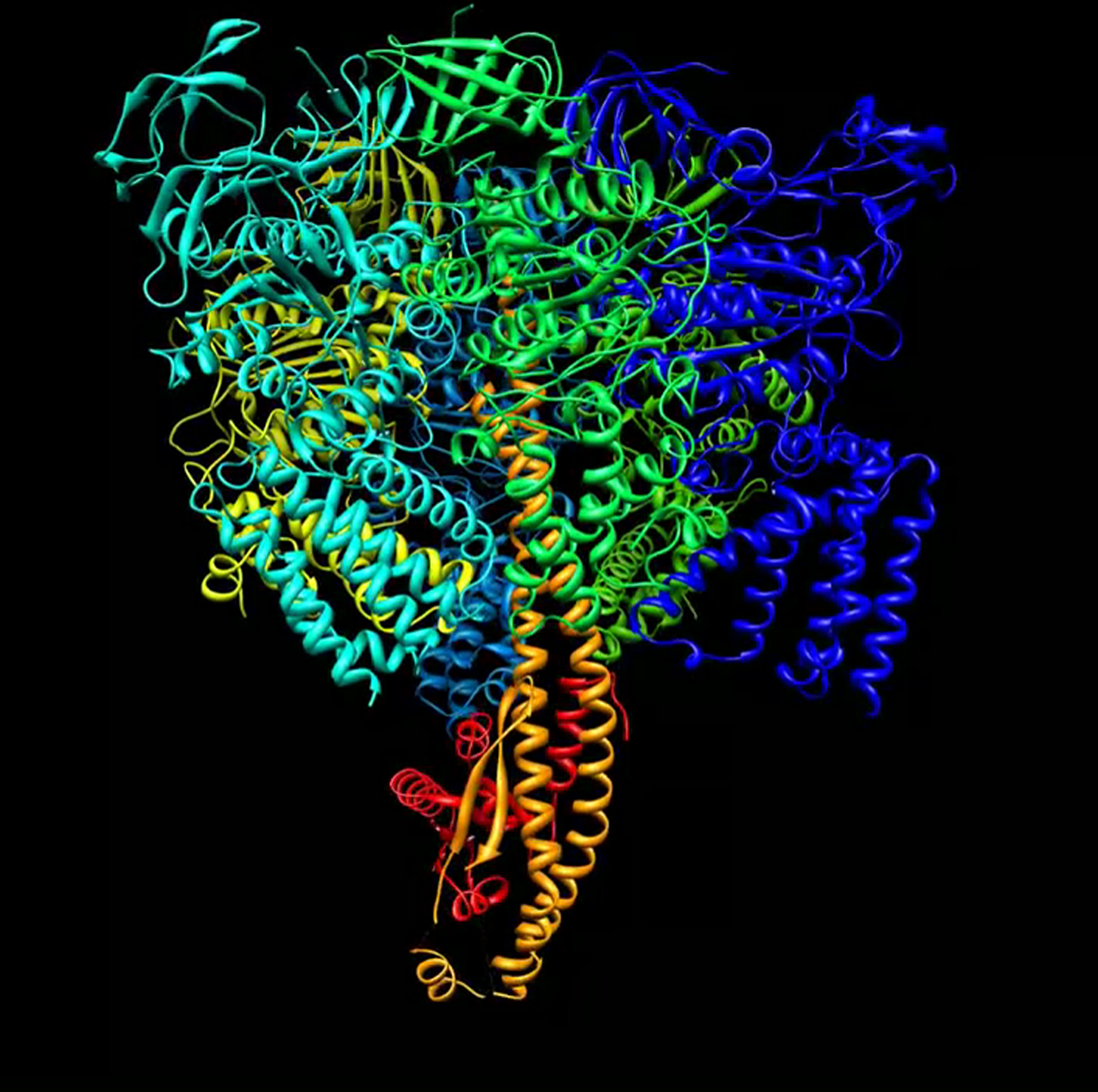

Supplement: Movie S2 (related to Figure 2). Structural Comparison between the A. hirae A1 and M. sexta V1 DomainThe two structures are morphed starting from the A. hirae structure and finishing with the M. sexta structure. Note the straightening of the central axle in the later structure [file mmc3.jpg]
